# Case of Letheon

**Published:** 1847-12

**Authors:** Asa Horr

**Affiliations:** Dubuque, Iowa


### ARTICLE V.

Dubuque, Iowa, Oct. 2, 1847.

Mr. Editor:

*Dear Sir*,—The following case is at your disposal.

A boy, 7 years old, was brought to me by his mother, three weeks ago, affected with amaurosis. It was thought advisable to have removed four much decayed molar teeth, when the lad, with his mother, accompanied me to the office of Dr. Lee, dentist, for the purpose. After much labor, the fangs of two teeth were skilfully removed—the patient displaying the utmost fortitude and self-possession.

This done, he entered his solemn protest against all further proceeding, and stoutly resisted argument, persuasion, and force. I proposed to control the case with the *letheon*, which was approved by the dentist, and by my partner, Dr. Sprague. A sponge, saturated with ether, was held to the nose in such a manner as to admit a due amount of atmospheric air. *To this he opposed all his strength*, but in about two minutes became quiet, then burst into an idiotic laugh, and soon passed into a tranquil sleep. During the repose, the other teeth were extracted, without the least manifestation of pain or consciousness—the sponge having been re-applied to check returning sensibility.

A few minutes after, he walked to our office, free from the effects of the gas, and ignorant of the latter part of the operation, which lasted ten or fifteen minutes. The letheon used was that commonly found in shops.

ASA HORR.

